# Comparing implicit boundary and hierarchical plurigaussian simulation for modeling geological domains using hard and soft data

**DOI:** 10.1038/s41598-026-55669-5

**Published:** 2026-06-03

**Authors:** Veronica Veliz, Jordan Plaza-Carvajal, Mohammad Maleki, Felipe Navarro, Eduardo Campos, Xavier Emery

**Affiliations:** 1 Department of Metallurgical and Mining Engineering, Universidad Católica del Norte, Antofagasta, Chile; 2 Advanced Mining Technology Center, Universidad de Chile, Santiago, Chile; 3 Department of Mining Engineering, Universidad de Chile, Santiago, Chile; 4 Department of Geological Sciences, Universidad Católica del Norte, Antofagasta, Chile

**Keywords:** Environmental sciences, Solid Earth sciences

## Abstract

The delineation of geological domains is a crucial step in mineral resource evaluation, as it directly influences the accuracy of grade models. Instead of a single geological interpretation, geostatistical simulation generates multiple scenarios that reproduce the spatial variability and can be used to assess uncertainty in the spatial layout of the geological domains. This study compares two established geostatistical simulation frameworks–Implicit Boundary Simulation (IBS) and Hierarchical Plurigaussian Simulation (HPGS)–and proposes practical, rigorous pathways to incorporate interpreted geological models as soft data. In IBS, the interpreted model enters as an auxiliary signed–distance covariate which is co-simulated with the down-the-hole signed distance derived from sampling data. In HPGS, the interpreted model informs local domain proportions through a moving window that controls local truncation thresholds. Both methods were applied to a porphyry copper deposit in northern Chile for modeling five mineralization zones under pronounced data scarcity. One hundred conditional realizations were generated for each approach and probability maps, most-probable domain maps, and match–mismatch tables against the interpreted model and drilling data were computed. The results demonstrated that, while both approaches benefited from incorporating soft data, HPGS yielded results that are more consistent with the interpreted model, particularly for mineralization zones exhibiting small-scale variability.

## Introduction

The spatial delineation of geological domains in the subsurface, such as rock types, mineralization, or alteration zones, is a critical step in mineral exploration and mining projects because it directly impacts the accuracy of mineral resource estimation. Geological domain modeling must provide a realistic representation of the deposit’s geological heterogeneity, which conditions grade models and mineral resource estimations^1–3^.

Implicit modeling^4^ has emerged in the past two decades as a powerful tool for geological modeling, due to its computational efficiency and versatility to represent varied geological settings. However, its inconveniences are twofold. On the one hand, it leads to a smooth spatial representation of the geological domains, hiding the true variability of the domain boundaries. On the other hand, it provides a single interpretation of the mineral deposit, which cannot be used to assess the uncertainty in the delineated domains.

Geostatistical simulation algorithms, including Sequential Indicator^5^, Multiple-Point^6^, Truncated Gaussian^7^, Plurigaussian^8^, and Implicit Boundary^9^ simulation, can be used for constructing geologically plausible representations of the domains and assessing the uncertainty in their boundaries. These methods generate multiple equally probable scenarios or “realizations” that reproduce the spatial variability inferred from sparse subsurface observations, allowing to incorporate uncertainty in geological domain modeling^10^ and to assess how this uncertainty propagates into the mineral resources, mineral reserves, and financial outcomes of mining projects^11–14^.

In practice, all the previously mentioned simulation algorithms face one common challenge that can result in geological models deviating from the actual characteristics of the deposit. This challenge relates to the quantity of data available for geological modeling. Indeed, the effectiveness of these algorithms is often constrained by the availability and spatial distribution of hard data, typically obtained from exploratory drill holes. In general, the amount of data is limited and insufficient compared to the spatial extent and geological heterogeneity of the mineral deposit.

To address this challenge, one solution is to incorporate a secondary source of information, such as an interpreted model of geological domains constructed based on geological knowledge of the deposit^15–17^. Although not directly measured, these models provide valuable spatial information and can be considered as soft data, helping to compensate for the lack of hard data in areas with a low sampling density. Despite their practical relevance, interpreted geological models are seldom fully integrated into geostatistical simulation workflows in a rigorous and systematic manner.

This study explores the integration of interpreted geological models into two established geostatistical simulation approaches: Implicit Boundary Simulation and Plurigaussian Simulation. The main objective is to assess how incorporating an interpreted geological model as a source of soft data influences the realism and consistency of simulated geological domains. We applied different strategies for each simulation approach to integrate the interpreted model and evaluate their impact on the resulting realizations. We aim to determine which approach better leverages the added value of geological expert knowledge in the presence of sparse hard data. This research is motivated by the pressing need to improve geological domain modeling under data scarcity conditions while preserving geological plausibility. By bridging the gap between geostatistical simulation techniques and expert-based interpretation, this work contributes toward more robust and informed geological models that enhance the reliability of downstream resource evaluations and inject uncertainty awareness into mineral exploration and mining project decisions.

The structure of this paper is as follows: section “Background” explains how the interpreted model of geological domains, used as a secondary source of information, can be integrated with drill hole data to reproduce the spatial behavior of geological domains in the aforementioned approaches. These methods are then applied to a porphyry copper deposit in section “Case study”. The results and discussions are presented in sections “Results” and “Discussion”, followed by the conclusions.

## Background

This section describes how an interpreted model of geological domains can be incorporated into the workflows of Implicit Boundary Simulation and Plurigaussian Simulation to enhance their performance under conditions of limited data availability.

### Implicit boundary simulation

Implicit Boundary Simulation^9^ is a hierarchical algorithm in which each level of the hierarchy (represented as a binary tree) involves simulating a geological domain and its complement. In this approach, the shortest distance to the contact between two geological domains ($V_{1}$) is calculated for each available sampling data. The simulation of the domain and its complement is then achieved by truncating simulated signed distances (with positive and negative values).

To address the challenge of limited data availability, an auxiliary covariate ($V_{2}$), derived from an interpreted model of the geological domains and available across the entire study area, is incorporated into the simulation process^17^. This covariate, like $V_{1}$, represents the shortest distance to the contact between domains. However, instead of being derived from the available sampling data, $V_{2}$ is calculated using the interpreted model of geological domains.

**Fig. 1:**
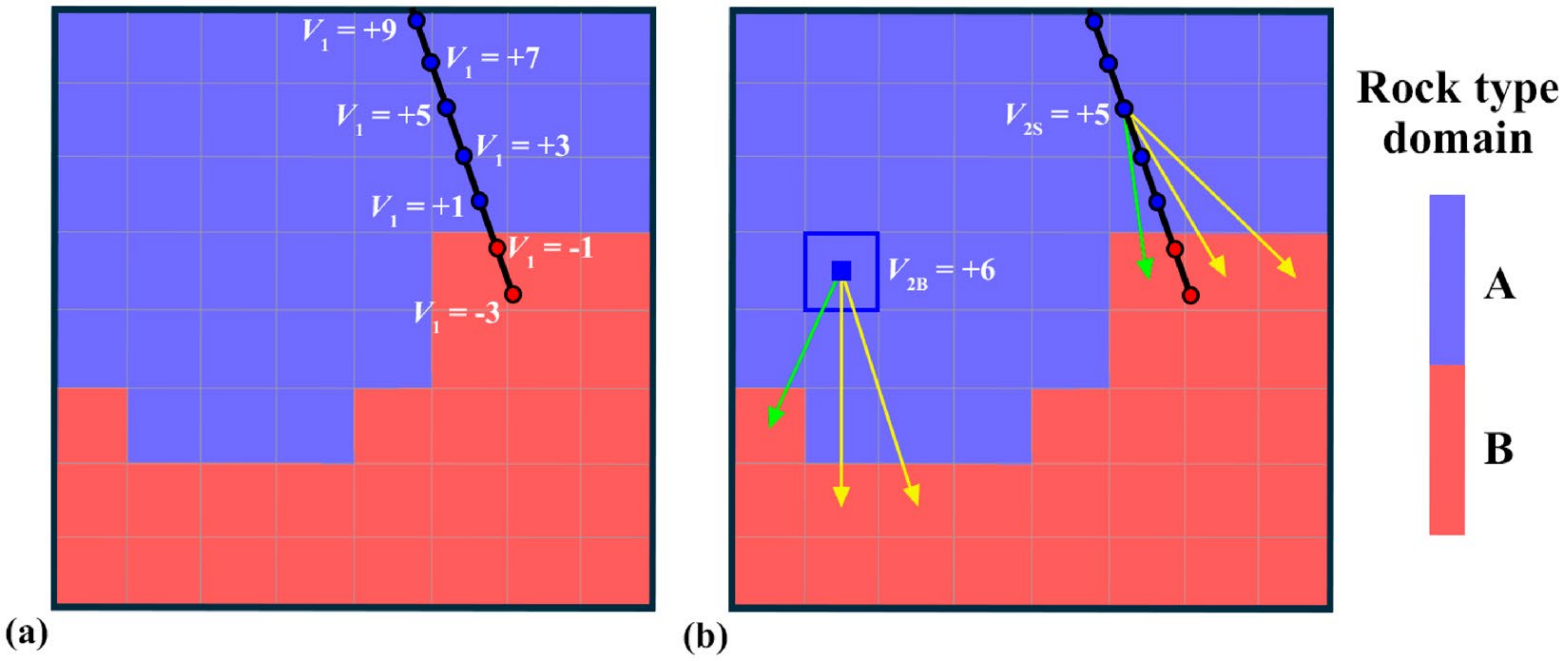
Graphical representation of the calculation of signed distances. **(a)** Distance from each sample to the nearest geological boundary along the same drill hole ($V_{1}$), and **(b)** distance from each sample and block to the nearest geological boundary (green arrows) in the interpreted geological model, corresponding to $V_{2S}$ and $V_{2B}$, respectively.

Additionally, while $V_{1}$ is only defined at the locations of hard data, $V_{2}$ is computed both at sample locations ($V_{2S}$) and across the entire block model ($V_{2B}$), providing a complete spatial coverage of the study area. Owing to its strong spatial correlation with $V_{1}$, the auxiliary variable $V_{2}$ serves as a valuable soft data source that compensates for the scarcity of hard data. This integration enhances the capacity of the simulation to reproduce domain structures and improves the overall reliability of the modeled geological realizations. Figure 1 illustrates the calculation process for each variable. As shown, $V_{1}$ is computed for each sampling data (represented by blue and red dots), while $V_{2}$ is determined not only at each sample location but also for every block in the interpreted model. It is worth noting that $V_{1}$ is calculated along the drill hole to which the sample belongs, whereas $V_{2}$ is calculated omnidirectionally, as depicted in Figure 1. It is important to clarify that $V_{1}$ is computed exclusively along the drill hole direction to ensure a precise and unambiguous definition of this variable. Consequently, the values remain unchanged regardless of the addition of new drill holes.

Following the above, $V_{1}$ and $V_{2}$ are transformed into standard Gaussian variables $Y_{1}$ and $Y_{2}$ through the application of transformation functions:

1 $$\begin{aligned} \begin{aligned} V_{1}&= \varphi _{1}\left( Y_{1} \right) \\ V_{2}&= \varphi _{2}\left( Y_{2} \right) , \end{aligned} \end{aligned}$$

where $\varphi _{1}$ and $\varphi _{2}$ are normal score transformation functions, $Y_{1}$ and $Y_{2}$ are the standard Gaussian values obtained by transforming the signed distances $V_{1}$ and $V_{2}$, respectively. These Gaussian variables can then be jointly simulated over the study area using any multi-Gaussian simulation algorithm^1^. By incorporating the covariate $Y_{2}$, which is fully known throughout the study area and highly correlated with the primary variable (partially known in the study area), the lack of information in the primary variable is compensated. This approach ensures that the realizations reproduce the behavior of geological domains, especially in areas with little to no sampling data, according to an interpretation based on geological expertise.

It is important to acknowledge that the geological interpretation, the primary source for calculating $V_{2}$, may not always be highly accurate. Due to this inherent uncertainty, it is essential to assign an appropriate level of confidence to the auxiliary variable $Y_{2}$ to prevent undue biases in the simulation outcomes. If an excessively high level of confidence is attributed to $Y_{2}$, it may dominate the simulation of the primary variable $Y_{1}$, particularly given the strong correlation between the two. To mitigate this effect, a scaling coefficient *ρ*, ranging from 0 to 1, is introduced. This coefficient is applied to the sill of the cross-variogram between the primary and auxiliary variables, thereby controlling the influence of $Y_{2}$ on the simulation results and allowing for a more balanced integration of the interpreted model.

**Fig. 2:**
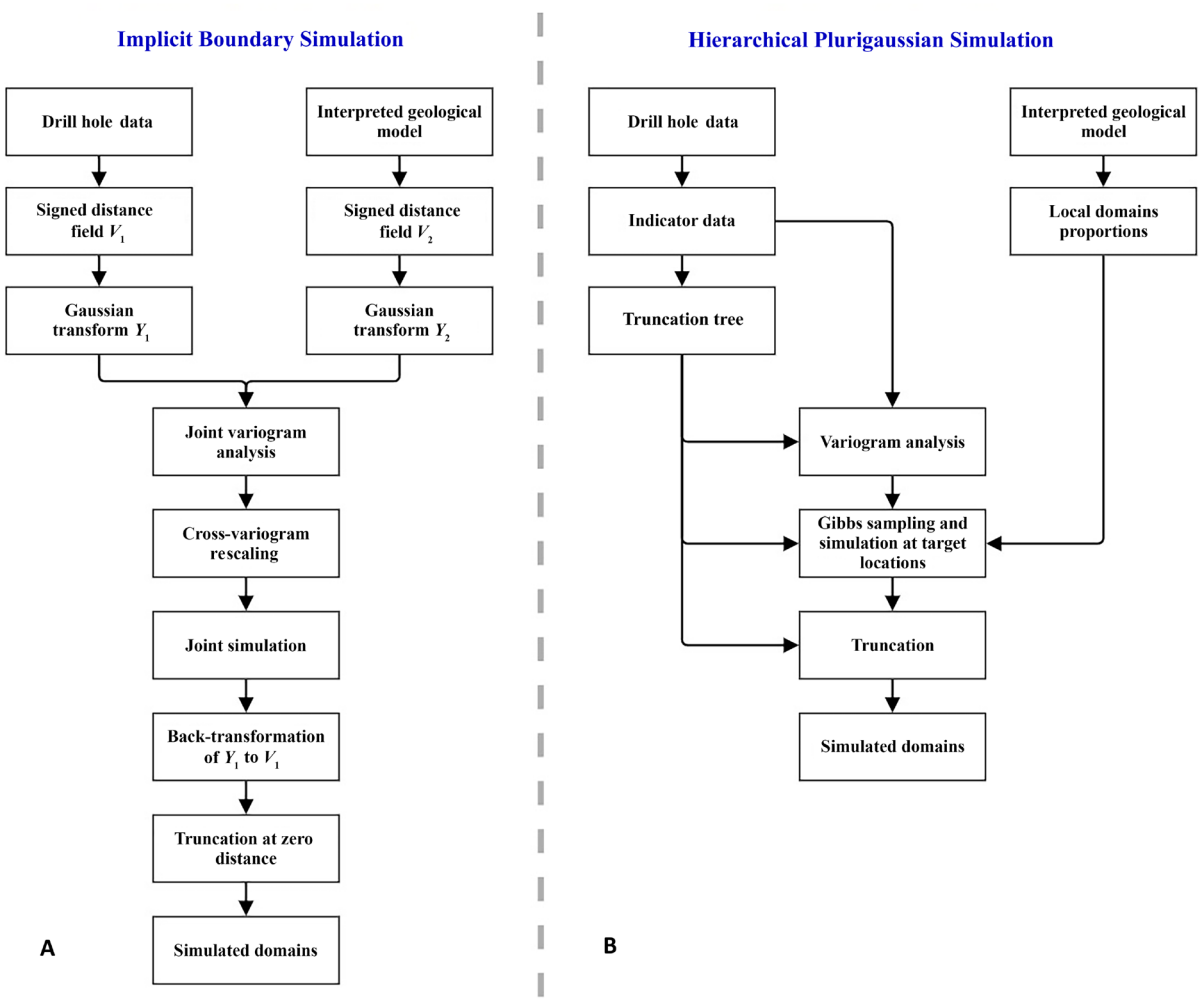
General workflow of (**A**) Implicit Boundary Simulation (IBS) and (**B**) Hierarchical Plurigaussian Simulation (HPGS). The IBS workflow from the definition of signed distance fields to the truncation step must be repeated for each level of the binary tree. The HPGS workflow from the variogram analysis to the truncation step must also be repeated for each level of the hierarchical truncation tree.

When the scaling coefficient is set to zero (*ρ = 0*), no correlation is assumed between the primary variable ($Y_{1}$) and the covariate ($Y_{2}$), so that the latter has no impact on the simulation of the former. Conversely, when *ρ* is set to one, the correlation between the two variables is fully preserved, allowing $Y_{2}$ to exert its maximum influence on the simulation of $Y_{1}$. The coefficient *ρ* therefore represents a “confidence level” that quantifies how much the interpreted geological model from which $V_2$ is derived can be trusted. To determine an appropriate value for *ρ*, a cross-validation-based approach can be employed, as detailed later^17^.

A summary of the main steps of Implicit Boundary Simulation is presented in Fig. 2A.

### Hierarchical Plurigaussian simulation

Hierarchical plurigaussian simulation^18,19^ is a variant of the plurigaussian simulation method in which, instead of signed distance fields as in Implicit Boundary Simulation, synthetic (latent) Gaussian random fields are simulated in a hierarchical sequence that can be represented through a truncation tree. This approach enables the reproduction of complex relationships between geological domains, as any number of Gaussian random fields can be incorporated as needed.

When sampling data are scarce, the true proportions of geological domains are uncertain, which can lead to the generation of realizations that are inconsistent with the actual characteristics of the mineral deposit^20^. An effective strategy to address this limitation is to use local domain proportions–derived from an interpreted model of geological domains–instead of relying on global proportions^19^. Since the interpreted model provides a complete spatial coverage of the study area, it allows for the estimation of local domain proportions around each location within the study area. These calculated local proportions are then used to determine local thresholds for truncating the simulated Gaussian random fields, as well as for converting the domain data (categorical) into Gaussian data via Gibbs sampling^10,21^.

**Fig. 3:**
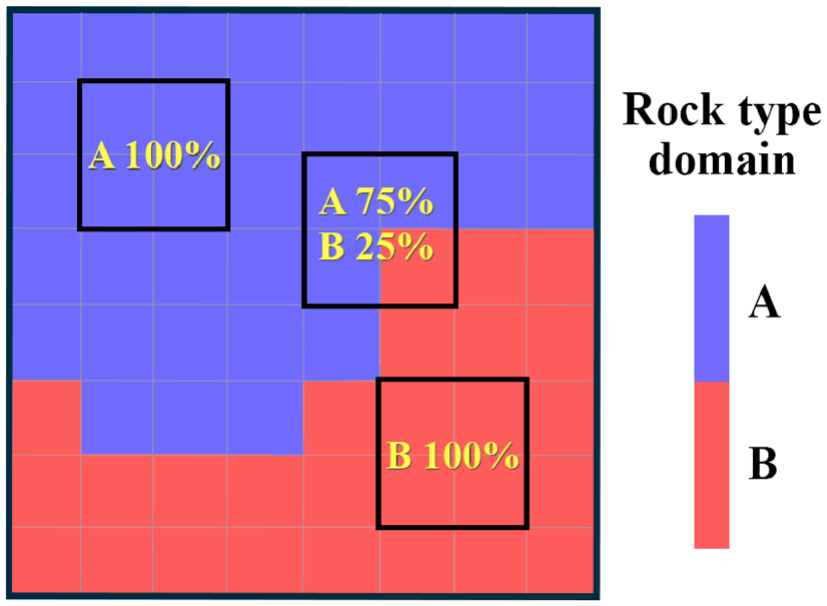
Graphical representation of local proportions calculation using a moving window.

To this end, a moving window–whose size is defined by the user–is applied to scan the entire interpreted model and compute the local proportions of geological domains at each location within the study area (Fig. 3). The key factor in this process is the size of the moving window, which directly impacts the quality of the results. If the window is too small, the generated realizations tend to closely replicate the interpreted model of geological domains, as the local proportions will predominantly be close to 0% or 100%, meaning complete certainty on the absence or presence of a domain. On the other hand, if the window is too large, the calculated local proportions of the domains approach the global proportions, making it challenging to reproduce the local behavior of the domains. The size of the moving window therefore plays the same role as the “confidence level” in Implicit Boundary Simulation, as it injects more or less confidence in the interpreted geological model into the hierarchical plurigaussian workflow. To determine the optimal size of the moving window, a cross-validation procedure^16,19^, similar to that used in the Implicit Boundary Simulation, can be conducted, as detailed in a later section.

A summary of the main steps of Hierarchical Plurigaussian Simulation is presented in Fig. 2B.

## Case study

In this section, Implicit Boundary Simulation and Plurigaussian Simulation are employed to model the mineralization zones of a porphyry copper deposit located in northern Chile (Atacama Desert). The area of interest for modeling is extensive, particularly in relation to the limited amount of available sampling data. This scarcity of hard data poses significant challenges for simulating the mineralization zones. To mitigate this limitation, an interpreted model of the mineralization zones is incorporated into the simulation process. For confidentiality reasons, the name and specific details of the mineral deposit are not disclosed.

### Mineralization zones

The Atacama Desert is globally recognized for hosting world-class porphyry copper deposits^22^. In these complex systems, ore mineralization is typically classified based on the origin of the ore minerals into three main zones: the hypogene zone (characterized primarily by chalcopyrite, bornite, and pyrite), the supergene enrichment zone (dominated by covellite and chalcocite), and the oxidized copper zone (comprising minerals such as chrysocolla, atacamite, and malachite)^23,24^. Hypogene sulfides are typically deposited at depths of approximately 5 km and are closely associated with magmatic-hydrothermal fluids derived from intermediate to silicic magmas^22,25^. As these hypogene sulfides are exhumed through tectonic uplift and erosion, descending meteoric waters induce chemical weathering in the vadose zone above the water table, resulting in the oxidation and leaching of sulfides^26–28^. The dissolved copper may subsequently be re-precipitated either as oxidized copper minerals above the water table or as supergene-enriched sulfides below it, depending on the conditions of precipitation^23,29^. This entire process leads to the development of a typical supergene profile, comprising an upper leached and oxidized zone underlain by a supergene copper-enriched blanket, which grades downward into the primary hypogene mineralization^23,26,27,30^. The investigated porphyry copper system displays a well-developed supergene weathering profile, within which the distribution of ore minerals is classified into five distinct mineralization zones, described as follows:

- Supergene Oxidized zone: dominated by minerals such as chrysocolla, malachite, and cuprite, it occurs closer to the surface and is an economically important source for leaching copper.
- Secondary Supergene Sulfide zone: characterized by the copper enrichment of hypogene sulfides, it typically contains chalcocite and covellite, formed just below the oxide zone as a result of supergene enrichment processes.
- Transitional Supergene Sulfides zone: underlying the Supergene Enriched Sulfide zone, it contains a mixture of hypogene and supergene sulfides. It often exhibits intermediate copper grades and plays a role in the overall metal budget of the deposit.
- Low-Grade Hypogene zone: it represents the outer parts of the hypogene system. This zone contains disseminated and vein-hosted mineralization consisting of chalcopyrite and pyrite. It typically exhibits lower copper grades compared to the high-grade hypogene zone.
- High-Grade Hypogene zone: localized near the core of the porphyry system, it is associated with the deepest parts of the deposit and characterized by high copper grades and mineralization dominated by chalcopyrite and bornite.

### Available data

**Fig. 4:**
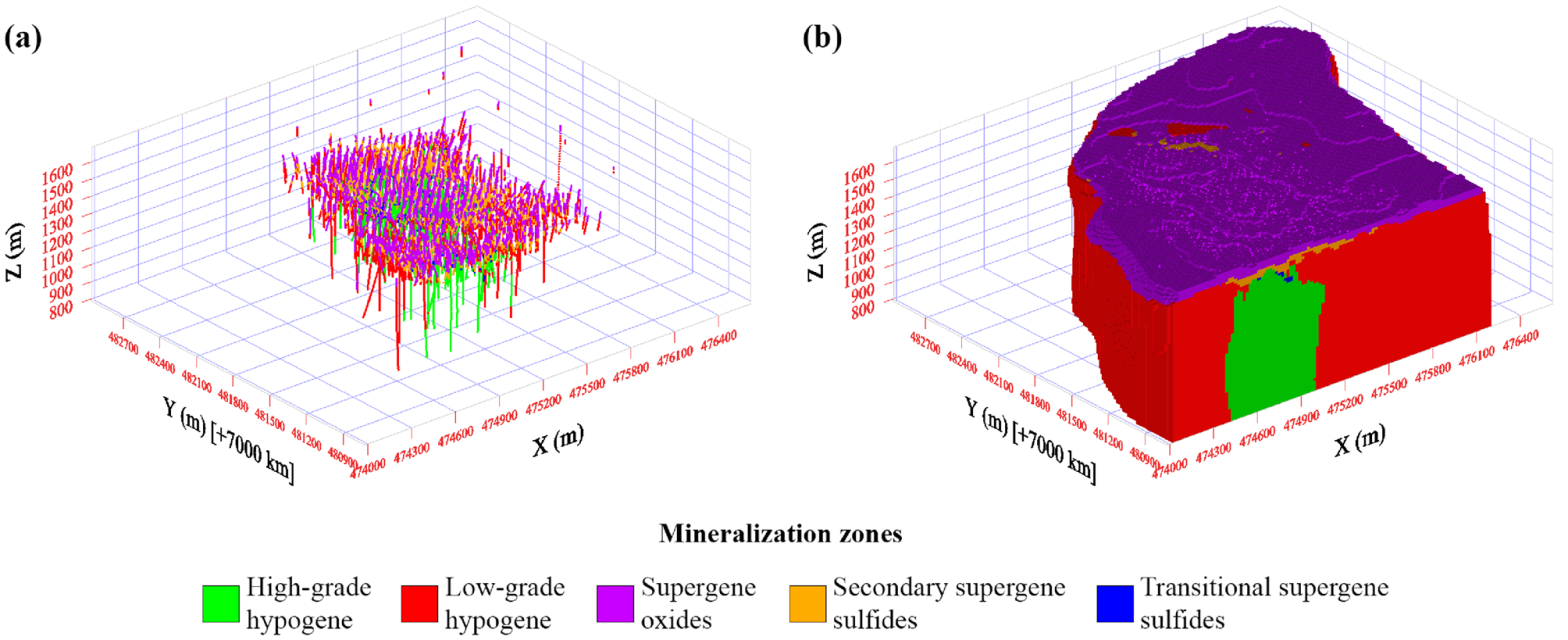
**(a)** Location of the exploratory drill holes (hard data) and **(b)** interpreted model of mineralization zones (soft data).

Two sources of information are available from this deposit. The first source is the exploratory drill hole data, comprising 1249 drill holes and a total of 67,050 samples. These samples provide detailed and quantitative information on five distinct mineralization zones, as shown in Fig. 4a. The second source is an interpreted model of the mineralization zones, constructed by expert geologists based on their comprehensive geological understanding of the deposit. This model is the result of an integrative interpretation process, in which geological knowledge–such as lithological contacts, structural trends, alteration patterns, and mineralization styles–has been synthesized to generate a spatially coherent representation of the subsurface. In essence, this interpreted model embodies the geologists’ conceptualization of the deposit, built upon years of field observations, prior data, and experience with similar geological settings. The model is discretized into blocks measuring $20\text {m} \times 20\text {m} \times 15\text {m},$ yielding a total of 583,175 blocks, as illustrated in Fig. 4b. As such, it serves as a valuable source of soft data, representing expert-derived knowledge that complements the sparse but precise hard data from drill holes.

Porphyry copper deposits commonly exhibit a pronounced vertical separation of mineralization zones^31,32^. For example, supergene minerals are located near the surface, formed through weathering and oxidation processes, while hypogene minerals occur at greater depths as a result of deeper geological processes^31^. This vertical separation is evident in this deposit, as observed in both the drill hole data and the interpreted deposit model, as shown in Fig. 4.

**Fig. 5:**
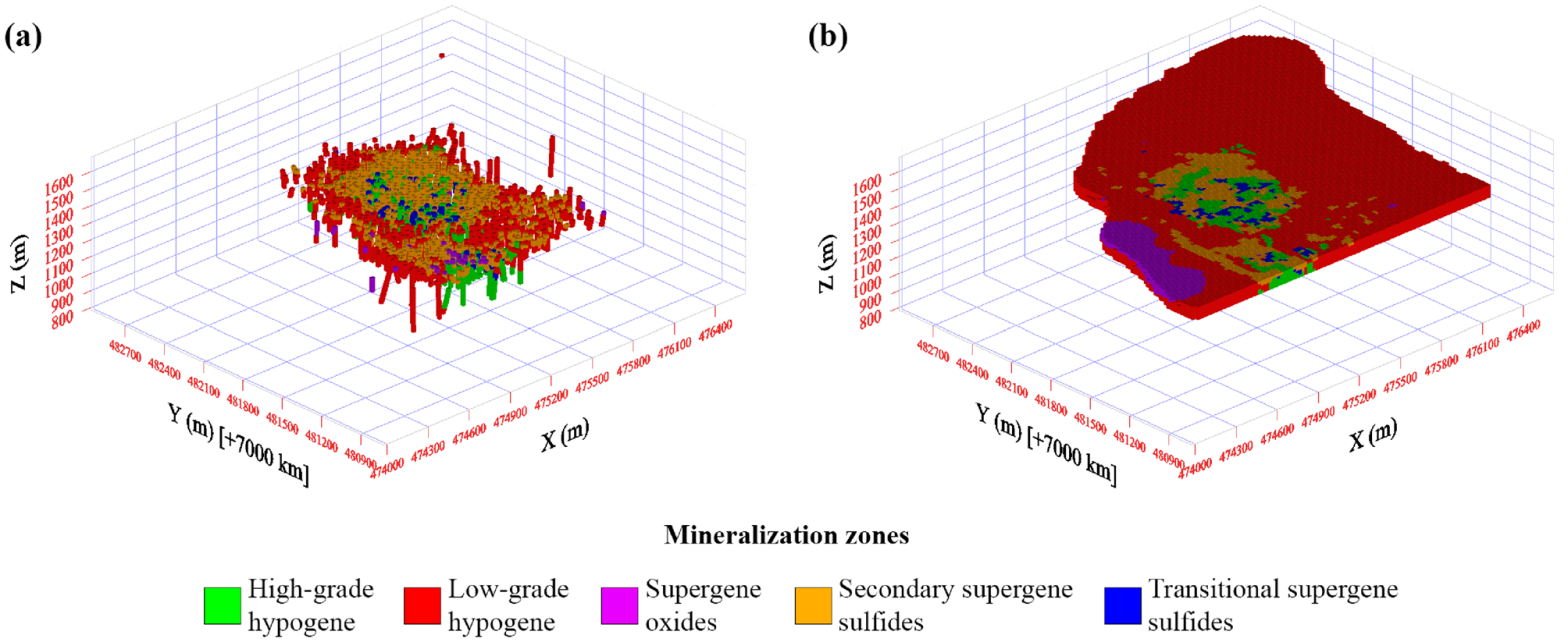
**(a)** Sampling data (hard data) and **(b)** interpreted model of mineralization zones (soft data) situated within the delimited section of the study area.

As depicted in Fig. 4, the area of interest for modeling is substantially larger than the number of available sampling data. In fact, certain parts of the study area lack any drill hole information, and the only source of geological insight in those regions is the interpreted model of mineralization zones. This situation highlights the critical importance of the interpreted model (the second source of information) in capturing the spatial distribution of geological domains. Its role becomes particularly essential in data-sparse areas, where direct observations are unavailable and the model serves as the primary basis for spatial inference. In this study, given the extensive size of the overall study area and the need to enhance computational efficiency, the simulation of the underlying mineralization zones was restricted to a smaller, representative subregion. This focused area was selected to balance geological complexity with computational feasibility, ensuring that the key spatial patterns and mineralization zones relationships are preserved. Figure 5 illustrates the distribution of the sampling data and the interpreted model of mineralization zones within this delimited section. The proportions of the different mineralization zones, as derived from both the drill hole data and the interpreted model within the selected area, are summarized in Table 1.

**Table 1:** Proportions of mineralization zones in drill hole data (hard data) and interpreted block model (soft data).

Mineralization zone	Proportions in hard data (%)	Proportions in soft data (%)
High-grade hypogene	32.22	10.69
Low-grade hypogene	39.48	81.08
Supergene oxides	0.81	2.67
Secondary supergene sulfides	23.55	4.24
Transitional supergene sulfides	3.94	1.33

### Implicit boundaries simulation

#### Hierarchical tree

**Fig. 6:**
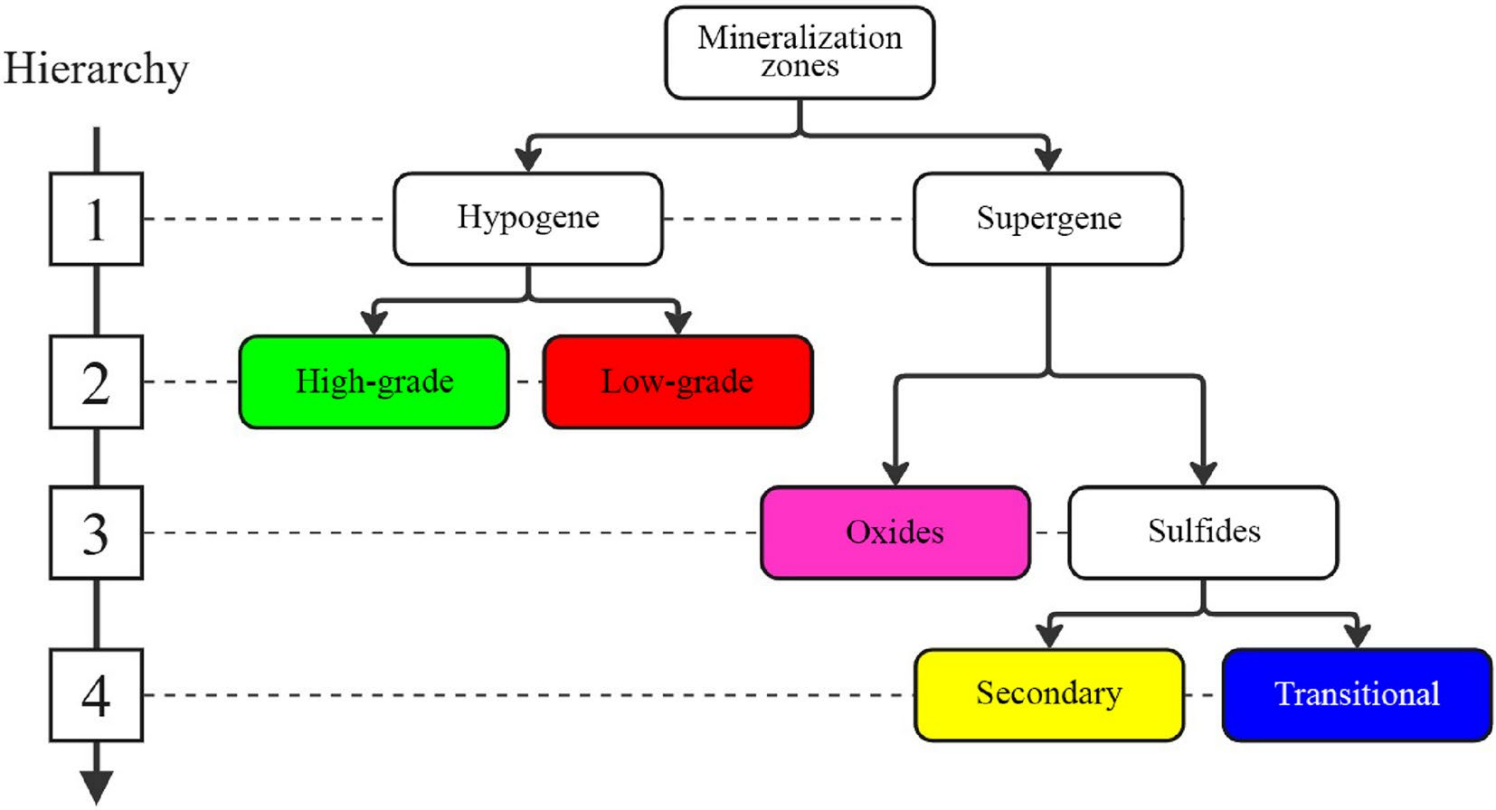
Hierarchical tree with four levels defined by expert geologists based on geological knowledge of the deposit, used to guide the implicit boundary simulation of mineralization zones. The first level separates hypogene and supergene zones; the second subdivides the hypogene into high-grade and low-grade domains; the third differentiates supergene oxides from supergene sulfides; and the fourth distinguishes secondary from transitional supergene sulfides.

To simulate the underlying mineralization zones using implicit boundary simulation, a hierarchical tree with four levels was defined and proposed by the expert geologists, based on the geological characteristics of the deposit and their in-depth knowledge of its formation and alteration processes. The first level distinguishes between hypogene and supergene zones–an essential division in porphyry systems that reflects the fundamental geological processes involved: primary mineralization at depth (hypogene) versus secondary enrichment and oxidation near the surface (supergene). At the second level, the hypogene domain is subdivided into high-grade and low-grade subzones. This division is motivated by economic and metallurgical considerations, as well as observed spatial patterns in grade distribution that typically reflect variations in hydrothermal fluid pathways and intensity of alteration. The third level focuses on the supergene zone, separating it into supergene oxides and supergene sulfides. This distinction is critical because these subdomains arise from different weathering mechanisms: oxidation in the vadose zone leads to the formation of oxides, while enrichment processes below the water table favor the precipitation of secondary sulfides. Finally, the fourth level further refines the supergene sulfide category by differentiating between traditional supergene sulfides and secondary supergene sulfides. This subtle distinction is based on variations in mineralogy, texture, and geochemical signatures that are relevant for processing and mineral resource evaluation. Figure 6 illustrates the complete hierarchical tree used in the implicit boundary simulation framework.

#### Joint spatial structure modeling

For each hierarchy, the signed distances ($V_{1}$ and $V_{2}$) were computed and subsequently transformed into their corresponding Gaussian variables ($Y_{1}$ and $Y_{2}$). The next step involved calculating the experimental omnidirectional direct and cross variograms of $Y_{1}$ and $Y_{2}$ for each hierarchy. A theoretical variogram model was then fitted to these experimental variograms.

**Fig. 7:**
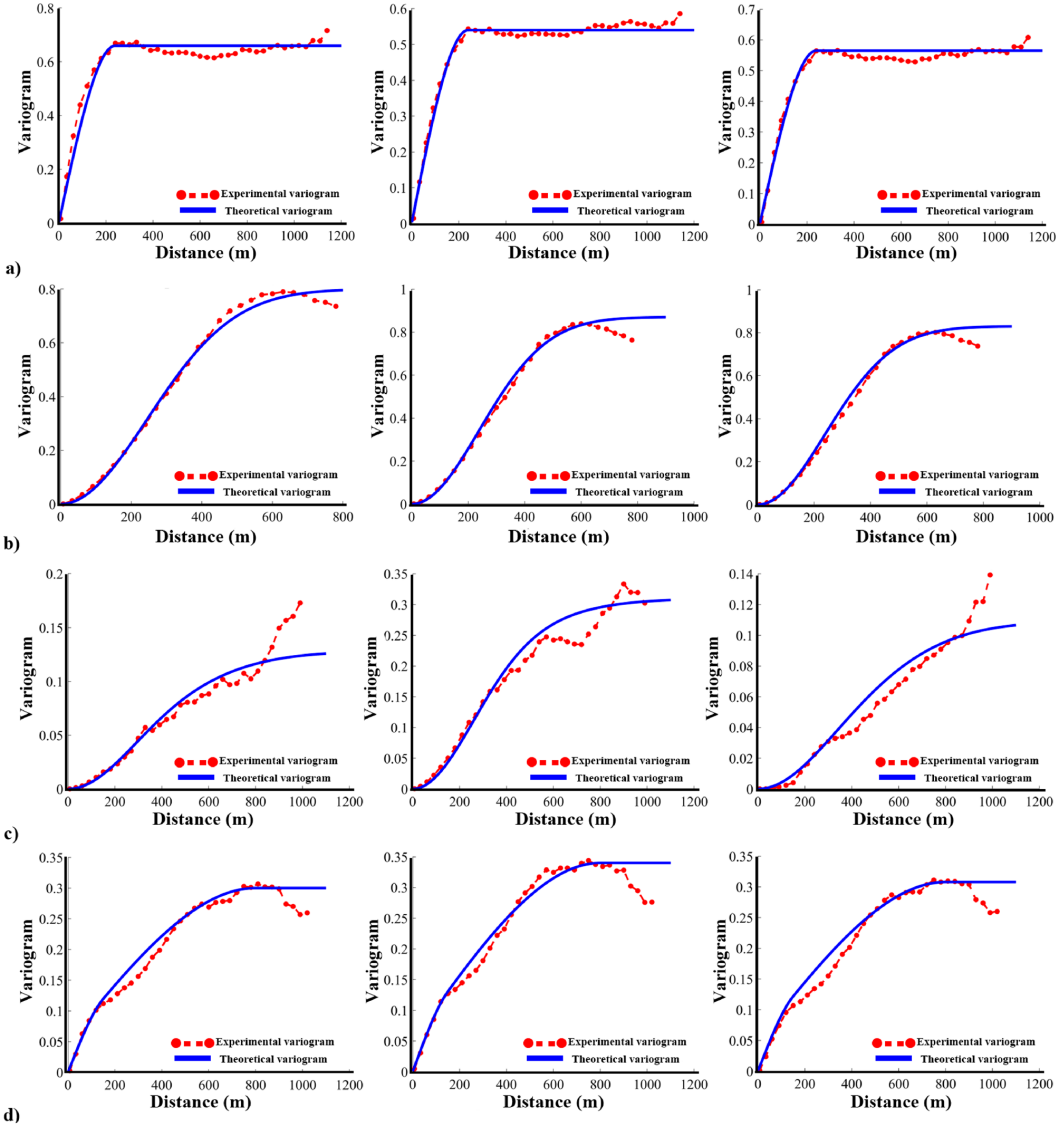
Experimental (red dashed line) and theoretical variogram models (blue solid line) for Implicit Boundary Simulation. Left: direct variogram of $Y_{1}$. Middle: direct variogram of $Y_{2S}$. Right: cross-variogram between $Y_{1}$ and $Y_{2S}$. This same order is repeated for each hierarchy: **(a)** hierarchy one, **(b)** hierarchy two, **(c)** hierarchy three, and **(d)** hierarchy four.

Due to the high density of nodes in the interpreted block model, the dataset for the second variable ($V_{2}$, omnidirectional distance to the boundary) is considerably larger than that of the first variable ($V_{1}$, down-the-hole distance to the boundary). Consequently, after transforming these variables into their Gaussian equivalents ($Y_{1}$ and $Y_{2}$), the joint variogram analysis is conducted using only the normal scores at the drill hole locations. This constraint is applied to prevent inconsistencies between the experimental direct and cross-variograms^33^. However, this approach results in direct variograms with sills significantly lower than the expected unit sill (Fig. 7). The primary cause of this discrepancy is the spatial clustering of drill hole data, which is predominantly concentrated in the central part of the deposit. As a result, these data do not fully capture the entire range of signed distances derived from the interpreted model. To address this limitation, the direct and cross-variograms of $Y_{1}$ and $Y_{2}$ are rescaled as follows:

2 $$\begin{aligned} \Gamma \left( h \right) = \begin{pmatrix} \gamma _{11}\left( h \right) \ & \gamma _{12}\left( h \right) \\ \gamma _{12}\left( h \right) & \gamma _{22}\left( h \right) \end{pmatrix} = \begin{pmatrix} \frac{\gamma _{11}\left( h \right) }{\gamma _{11}\left( \infty \right) }& \frac{\gamma _{12}\left( h \right) }{\sqrt{\gamma _{11}\left( \infty \right) \gamma _{22}\left( \infty \right) }} \\ \frac{\gamma _{12}\left( h \right) }{\sqrt{\gamma _{11}\left( \infty \right) \gamma _{22}\left( \infty \right) }} & \frac{\gamma _{22}\left( h \right) }{\gamma _{22}\left( \infty \right) } \end{pmatrix}, \end{aligned}$$

where $\gamma _{ij}$ *(i,j=1,2)* represent the original, non-rescaled variograms, $\gamma _{ij}(\infty )$ denote their respective sills, and *Γ (h)* correspond to the matrix of rescaled variograms. This rescaling process ensures that the two Gaussian random fields, $Y_{1}$ and $Y_{2}$, achieve a unit sill, thereby maintaining unit variance.

The resulting fitted models for each hierarchy are represented by Eq. (3), which use isotropic spherical (Sph) and Gaussian (Gauss) structures:

3 $$\begin{aligned} \begin{aligned} \gamma _{H1}(h)&= \begin{pmatrix} \gamma _{1}(h) & \gamma _{12}(h) \\ \gamma _{12}(h) & \gamma _{2}(h) \end{pmatrix} = \begin{pmatrix} 0.660 & 0.565 \\ 0.565 & 0.540 \end{pmatrix} \text {Sph}(240 \, \text {m}) \\ \\ \gamma _{H2}(h)&= \begin{pmatrix} \gamma _{1}(h) & \gamma _{12}(h) \\ \gamma _{12}(h) & \gamma _{2}(h) \end{pmatrix} = \begin{pmatrix} 0.80 & 0.85 \\ 0.85 & 0.88 \end{pmatrix} \text {Gauss}(600 \, \text {m}) \\ \\ \gamma _{H3}(h)&= \begin{pmatrix} \gamma _{1}(h) & \gamma _{12}(h) \\ \gamma _{12}(h) & \gamma _{2}(h) \end{pmatrix} = \begin{pmatrix} 0.05 & 0.02 \\ 0.02 & 0.22 \end{pmatrix} \text {Gauss}(600 \, \text {m}) + \begin{pmatrix} 0.078 & 0.089 \\ 0.089 & 0.090 \end{pmatrix} \text {Gauss}(1000 \, \text {m})\\ \\ \gamma _{H4}(h)&= \begin{pmatrix} \gamma _{1}(h) & \gamma _{12}(h) \\ \gamma _{12}(h) & \gamma _{2}(h) \end{pmatrix} = \begin{pmatrix} 0.05 & 0.05 \\ 0.05 & 0.05 \end{pmatrix} \text {Sph}(150 \, \text {m}) + \begin{pmatrix} 0.250 & 0.258 \\ 0.258 & 0.290 \end{pmatrix} \text {Sph}(800 \, \text {m}). \end{aligned} \end{aligned}$$

Here, $\gamma _{1}(h)$, $\gamma _{2}(h)$, and $\gamma _{12}(h)$ represent the sills of the direct and cross variograms for $Y_{1}$ and $Y_{2}$, respectively. The numbers in brackets indicate the range of the direct and cross-variograms. The results of the direct and cross-experimental variograms, along with the theoretical models fitted for each hierarchy, are graphically presented in Fig. 7.

It is acknowledged that the last points of the experimental variogram shown in Figure 7c may visually suggest a possible large-scale trend. However, these points were calculated from a substantially smaller number of data pairs than those associated with shorter lag distances, reducing their statistical robustness. For this reason, they were not considered representative of the underlying spatial continuity structure, and the fitted variogram model was selected to reach a stable sill, in accordance with the assumption of second-order stationarity.

#### Choice of confidence coefficient *ρ*

As previously discussed, the sill of the cross-variograms quantifies the spatial dependency between $V_{1}$ (derived from the sampling data) and $V_{2}$ (derived from the interpreted model). A high correlation between these two variables is expected, given that the sampling data (hard data) were used–alongside other geological information–in the construction of the interpreted model of mineralization zones. However, in cases where there is low confidence in the interpreted model, and more freedom is desired for the realizations to deviate from the interpreted model, the sill of the cross-variogram can be multiplied by a coefficient (*ρ*) to reduce this dependency. In this study, following Ferrer et al.^17^, a cross-validation process was performed to define the optimum value of this coefficient.

To this end, 100 realizations of the mineralization zones were generated at each drill hole location, conditioned on both the interpreted geological model and neighboring drill hole data within a specified search radius. To prevent excessively strong local influence, data located within 50 meters of the target drill hole were excluded from conditioning. Following the simulations, the probability of occurrence for each mineralization zone was computed at all drill hole locations. Among all the drill hole data having the same probability value *P* for a specific mineralization zone, it is expected that approximately a proportion *P* of the drill hole data will correspond to that zone^17,19^. For instance, among all the data with a calculated probability of occurrence of 50% for the supergene domain, it is expected that half of these data would belong to this domain. This relationship between occurrence probabilities and data proportions can be evaluated for any probability *P* and any mineralization zone. Consequently, the impact of varying *ρ* was assessed within the simulation algorithm, and the optimal *ρ* value was determined.

**Fig. 8:**
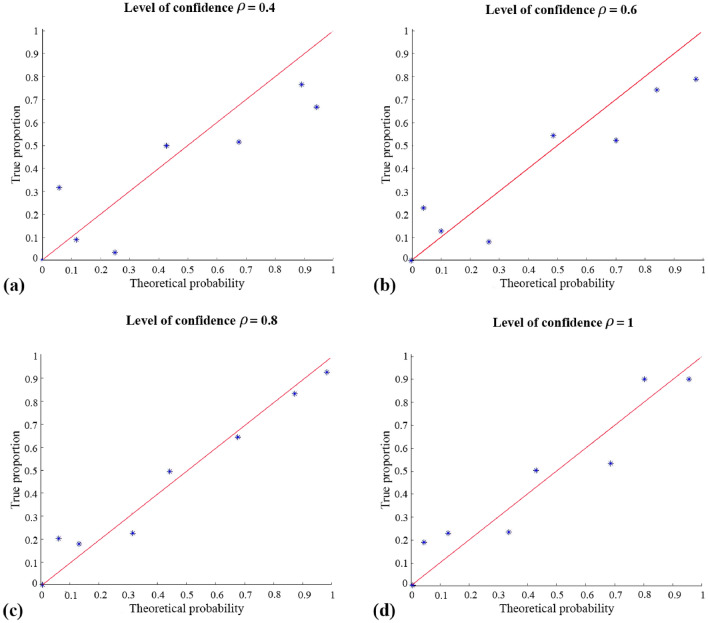
Results of the cross-validation process for the high-grade hypogene, used to determine the optimal value for *ρ*.

Figure 8 presents the results of cross-validation for the high grade hypogene (the second level of the binary tree). As shown, *ρ =0.8* is an optimal value for *ρ*, as it generates an accurate simulated model for the supergene zone (Fig. 8c), while more mismatches between theoretical probabilities and actual data proportions are observed when choosing lower values of *ρ* (Fig. 8a and b). Moreover, the simulated supergene zones align well with the interpreted model, especially in regions with sparse or no drill hole data. The same value of *ρ* was determined for the other mineralization zones. Consequently, *ρ =0.8* was selected as the optimal value at each level of the hierarchy.

#### Simulation of mineralization zones

**Fig. 9:**
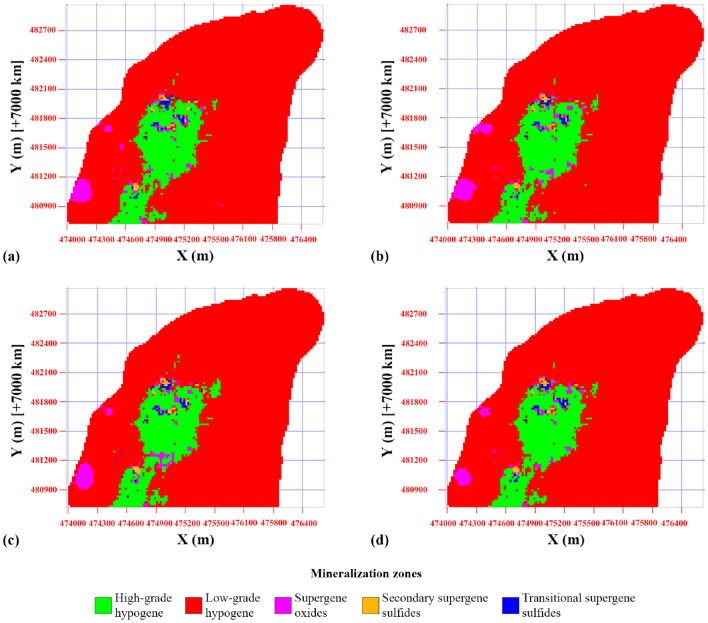
Four realizations of mineralization zones generated using the implicit boundary simulation approach, incorporating the interpreted geological model as a secondary source of information. Plan views at elevation 1522 m.

Once all the key parameters were defined, a continuous spectral simulation method^34^ was applied to jointly simulate the Gaussian variables $Y_{1}$ and $Y_{2}$ over a regular grid composed of $131 \times 111 \times 4$ nodes, with cell dimensions of $20\text {m} \times 20\text {m} \times 15\text {m}$–matching the resolution of the interpreted geological model. For each level of the hierarchical structure, one hundred conditional realizations were generated and subsequently back-transformed to the original signed distance variables, $V_{1}$ and $V_{2}$, using the respective transformation functions. The binary geological domains at each level of the hierarchy were then delineated by truncating the simulated signed distances at zero, effectively defining the domain boundaries. Following the hierarchical structure outlined in Fig. 6, these binary realizations were progressively combined to produce one hundred realizations of the five mineralization zones. Figure 9 presents four realizations of the resulting mineralization zones generated through the Implicit Boundary Simulation approach. The complete simulation workflow was implemented using in-house Python scripts (version 3.14.3), developed for this study.

### Hierarchical Plurigaussian simulation

#### Truncation tree and Gaussian variograms

The mineralization zones were also modeled using the Hierarchical Plurigaussian Simulation algorithm. The first step in this process involved defining an appropriate truncation tree. Based on the spatial relationships observed in the interpreted model and supported by the drill hole data, it was determined that all five mineralization zones were in mutual contact.

**Fig. 10:**
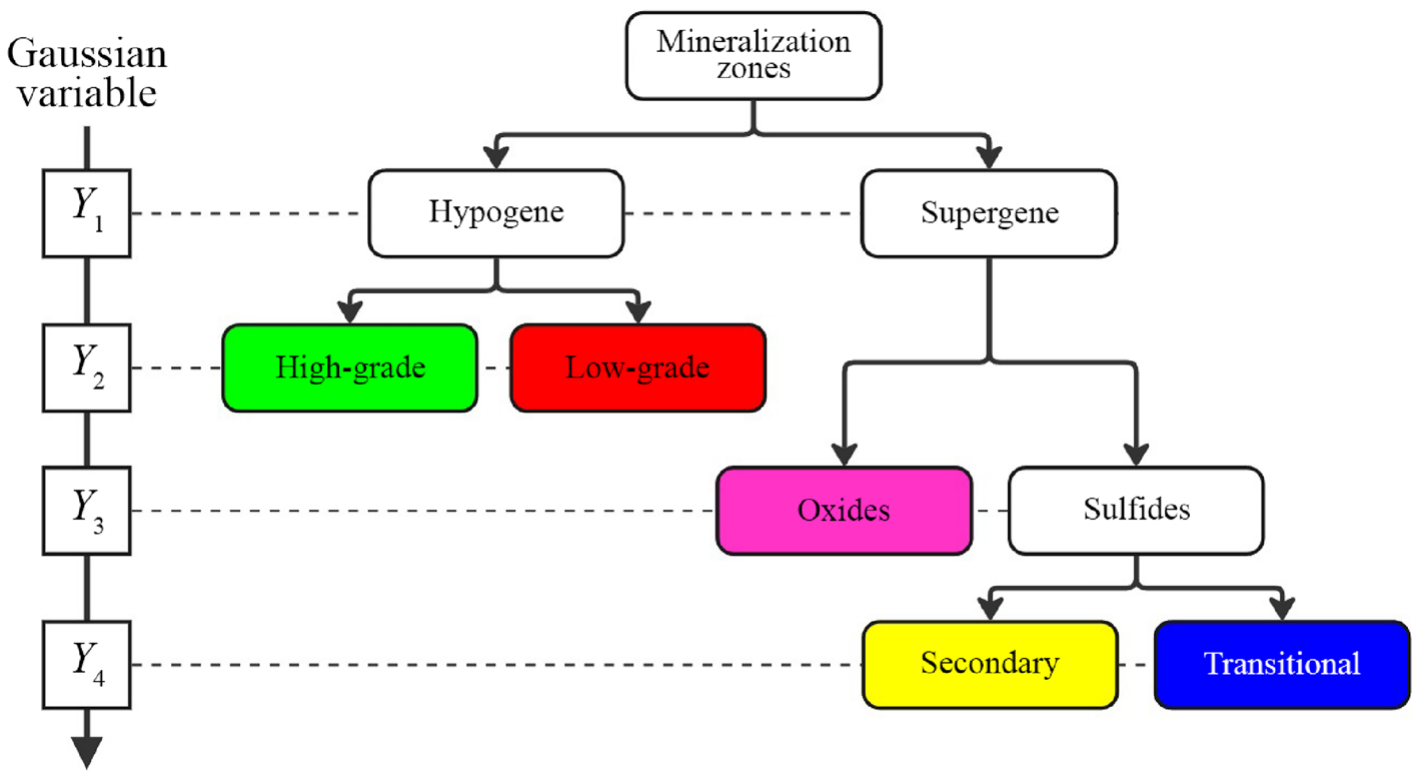
Truncation tree applied in hierarchical plurigaussian simulation with four latent Gaussian random fields.

Accordingly, the truncation tree was constructed to respect the admissible geological contacts among the mineralization zones, as illustrated in Fig. 10. This tree was defined using four independent standard Gaussian random fields ($Y_1$ to $Y_4$) and four truncation thresholds ($t_1$ to $t_4$), which collectively enabled the simulation of five mutually contacting domains, and can be mathematically formalized through Eq. (4):

4 $$\begin{aligned} \text {Mineralization zone at location } x = \left\{ \begin{array}{ll} \text {High-grade hypogene}, & \text {if } Y_1(x)< t_1 \text { and } Y_2(x)< t_2 \\ \text {Low-grade hypogene}, & \text {if }Y_1(x)< t_1 \text { and } Y_2(x) > t_2 \\ \text {Supergene oxides}, & \text {if } Y_1(x) \ge t_1 \text { and } Y_3(x)< t_3 \\ \text {Secondary supergene sulfides}, & \text {if } Y_1(x) \ge t_1, Y_3(x) \ge t_3\text { and } Y_4(x) < t_4\\ \text {Transitional supergene Sulfides}, & \text {if } Y_1(x) \ge t_1, Y_3(x) \ge t_3\text { and } Y_4(x) \ge t_4. \end{array} \right. \end{aligned}$$

The first Gaussian field, $Y_1$, governs the separation between the hypogene and supergene zones. Within the hypogene zone, a second Gaussian field, $Y_2$, is employed to distinguish between high-grade and low-grade subdomains. For the supergene zone, the third Gaussian field, $Y_3$, delineates the boundary between supergene oxides and supergene sulfides. Finally, the fourth Gaussian field, $Y_4$, further partitions the supergene sulfides into secondary sulfides and traditional sulfides. This hierarchical truncation strategy enables the consistent modeling of nested geological relationships while preserving geological realism.

We draw the reader’s attention to the similarity between the truncation tree in Fig. 10 and the hierarchical tree used in Implicit Boundary Simulation (Fig. 6). The two simulation approaches rely on the same geological conceptualization of the deposit and the same contact relationships between mineralizations zones, the difference being that Implicit Boundary Simulation works with signed distance random fields, whereas Hierarchical Plurigaussian Simulation works with latent Gaussian random fields.

**Fig. 11:**
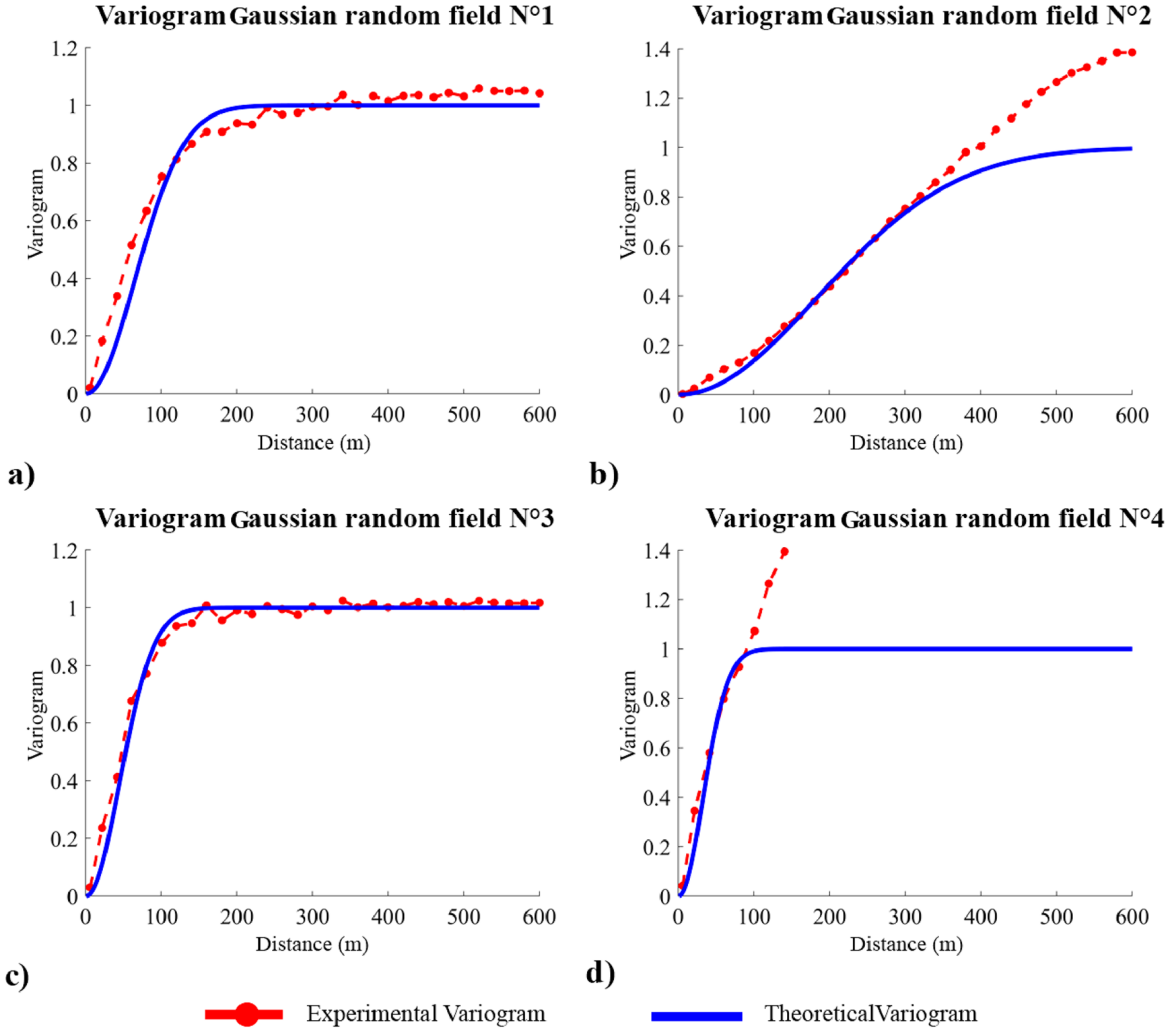
Experimental variograms (dashed lines) and theoretical variogram models (solid lines) for each Gaussian random field.

Following the definition of the truncation tree, the next step was to determine the spatial structure of each underlying Gaussian random field. To this end, the experimental indicator variograms for each domain were computed and subsequently transformed into variograms of the underlying Gaussian random fields, following the approach described by Emery^20^. The experimental and fitted theoretical variograms of the Gaussian random fields are presented in Fig. 11, while the corresponding variogram models are detailed in Eq. (5).

5 $$\begin{aligned} \begin{aligned} \gamma _{1}&= Gauss(80\,\text {m}, 80\,\text {m},80\,\text {m}) \\ \gamma _{2}&= Gauss(420\,\text {m}, 420\,\text {m},420\,\text {m}) \\ \gamma _{3}&= Gauss(100\,\text {m}, 100\,\text {m},100\,\text {m}) \\ \gamma _{4}&= Gauss(100\,\text {m}, 100\,\text {m},100\,\text {m}). \end{aligned} \end{aligned}$$

Note that the fitted models reach a unit sill, which is consistent with the plurigaussian model that assumes each Gaussian random field to be stationary and to have a unit variance, in particular for the definition of the truncation thresholds and for the indicator-to-Gaussian variogram transformation. Discrepancies between the experimental and fitted variograms are expected at large distances^1^ and therefore not a concern. Trends in the spatial distribution of the mineralization zones, which could explain that experimental variograms exceed the theoretical unit sill, will be captured via the consideration of the local proportions of each zone, as explained next.

#### Calculation of local domain proportions

As in Implicit Boundary Simulation, the interpreted model of mineralization zones, used as a secondary source of information, serves as a valuable tool for addressing the issue of sparse data. Specifically, a moving window is applied to scan the interpreted model and estimate the local proportions of mineralization zones throughout the study area. In the absence of an interpreted model, local proportions cannot be estimated, and only global proportions are used for simulating the mineralization zones. As a result, in areas with few or no hard data, any mineralization zone may appear in the simulated model, leading to geologically unrealistic outcomes that may be entirely inconsistent with the known characteristics of the deposit (e.g., supergene oxidized zone appearing at depth, or high-grade hypogene zone near the surface).

In this context, as previously emphasized, defining the optimal size of the moving window is a critical step for calculating the local proportions of mineralization zones, as it has a direct influence on the reliance on the interpreted geological model and on the quality of the simulation results. To determine this optimal size, a cross-validation procedure was conducted, following the approach described in earlier literature^16,19^.

**Fig. 12:**
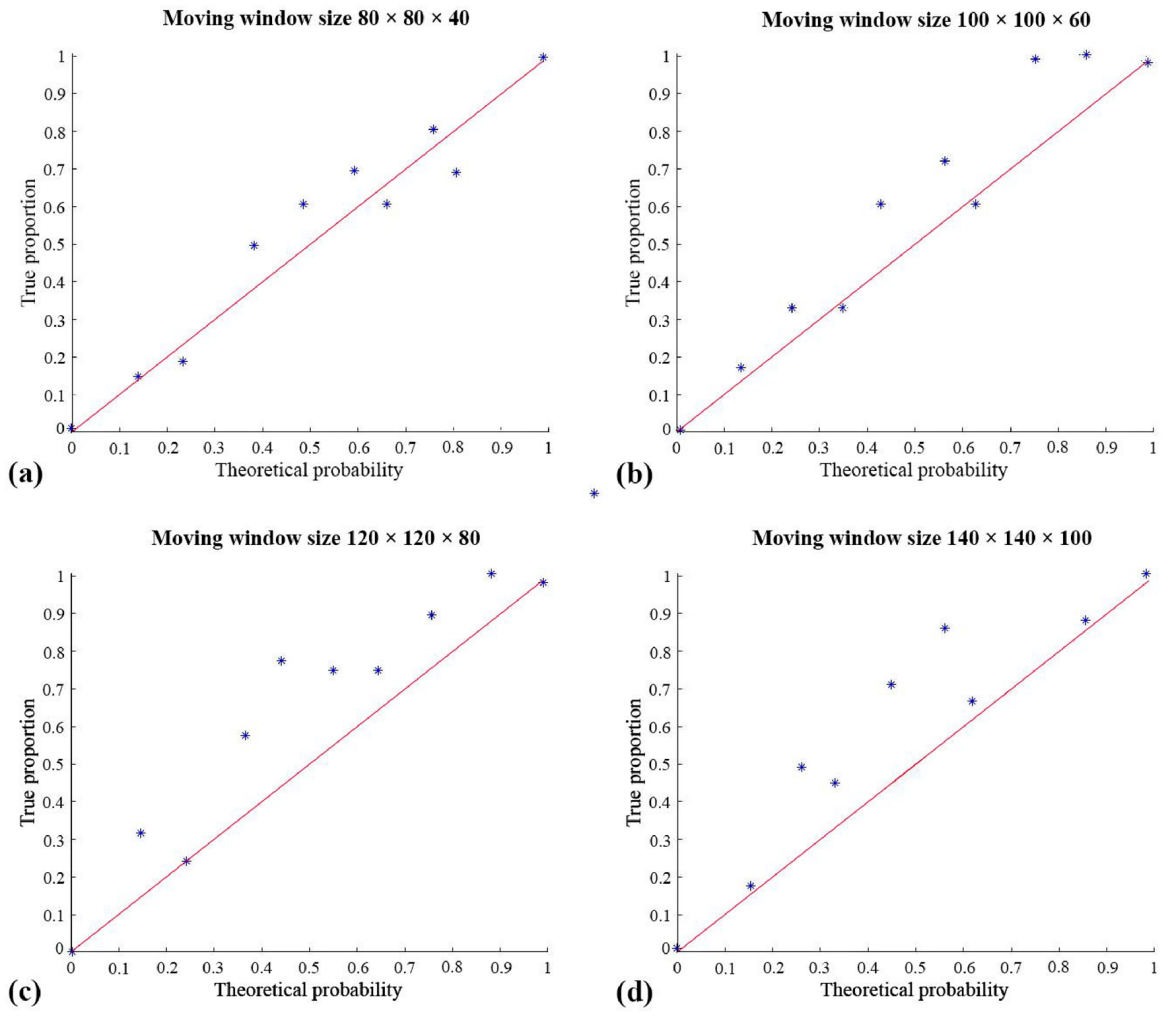
Results of the cross-validation procedure for the high-grade hypogene, used to determine the optimal size of the moving window for calculation of local proportions in the plurigaussian simulation framework.

For this purpose, the sampling dataset was partitioned into two subsets: a training set and a testing set. At each location in the testing set, 100 conditional realizations were generated using only the information contained in the training set. The estimated occurrence probabilities for the mineralization zones were then compared with the actual observed zones at the testing locations—that is, the same procedure as the one used in Implicit Boundary Simulation. This validation was repeated for multiple window sizes, across different theoretical probability intervals, and for each mineralization zone. The results of this analysis are presented in Figure 12 for the high-grade hypogene, illustrating the sensitivity of simulation performance to the choice of moving window size. As shown in the figure, a window size of $80\text {m} \times 80\text {m} \times 40\text {m}$ yields the most reliable results for capturing the uncertainty in the output models, providing a better match between predicted probabilities and observed occurrences. Consequently, these dimensions were used to calculate the local proportions of mineralization zones within the study area.

### Simulation of mineralization zones

**Fig. 13:**
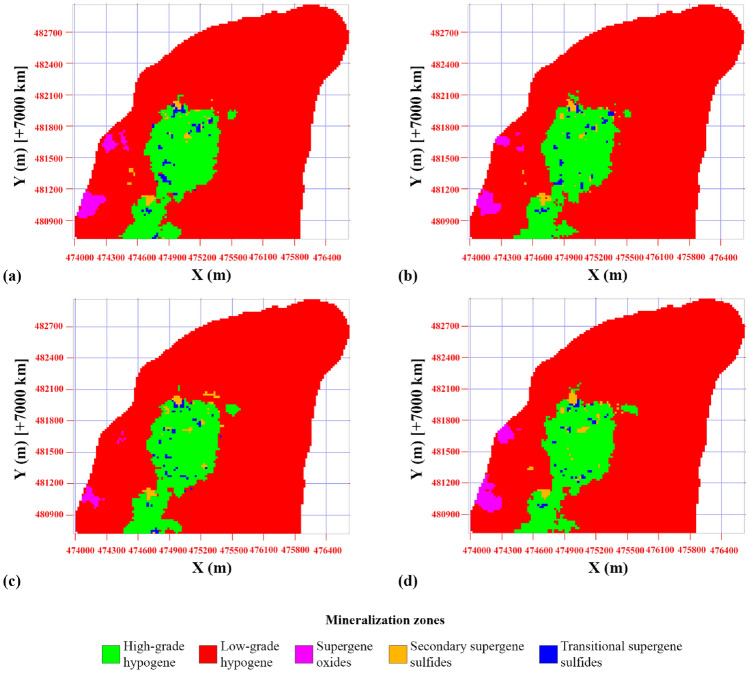
Four different generated realizations of mineralization zones using plurigaussian simulation. Plan views at elevation 1522 m.

After defining the key parameters, including the truncation tree, local domain proportions and variogram models for the latent Gaussian random fields, these fields were simulated across the study area and truncated based on the defined tree and local proportions, resulting in 100 realizations of the mineralization zones. Figure 13 illustrates four generated realizations using the Hierarchical Plurigaussian Simulation approach. Simulation was performed using in-house Matlab (R2023b) scripts.

## Results

**Fig. 14:**
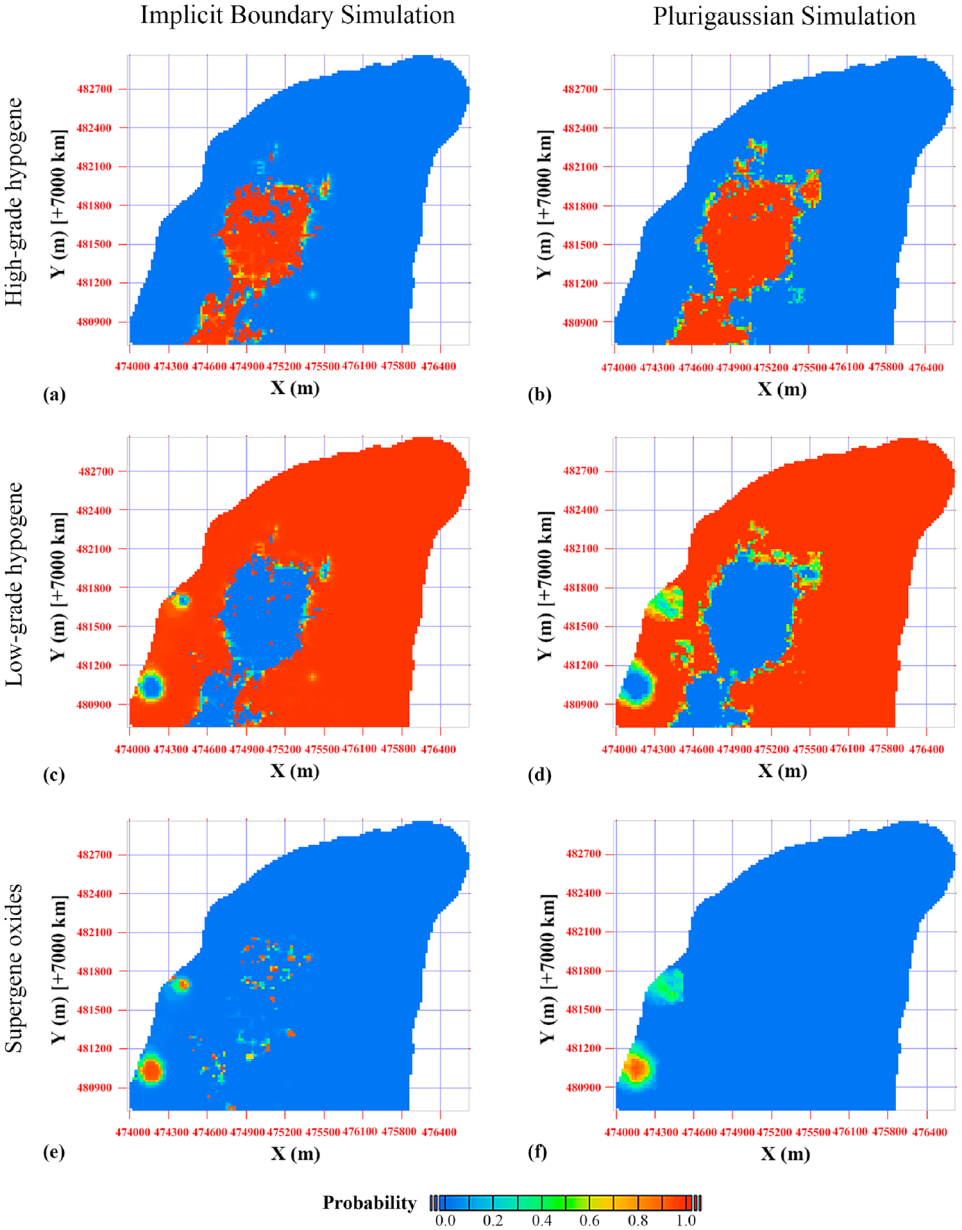
Probability maps of occurrence of three of the five mineralization zones obtained from Implicit Boundary Simulation and Hierarchical Plurigaussian Simulation. Plan views at elevation 1522 m.

Using 100 realizations generated from both approaches, the probability of occurrence for each mineralization zone was calculated across the study area. Figure 14 presents the probability maps for low-grade hypogene, high-grade hypogene, and supergene oxides derived. A visual comparison reveals a high degree of similarity between the two approaches for the probability maps of low-grade and high-grade hypogene zones. Specifically, both approaches indicate a higher probability of low-grade hypogene zones along the periphery of the study area, while high-grade hypogene zones are more likely concentrated in the central region. In contrast, the probability maps for supergene oxides exhibit notable differences (Fig. 14e and f). While both approaches lead to a high probability of supergene oxides on the left side of the study area, the Implicit Boundary Simulation also predicts a significant probability in the central region. This prediction contradicts the interpreted mineralization model and established geological knowledge of the deposit.

**Fig. 15:**
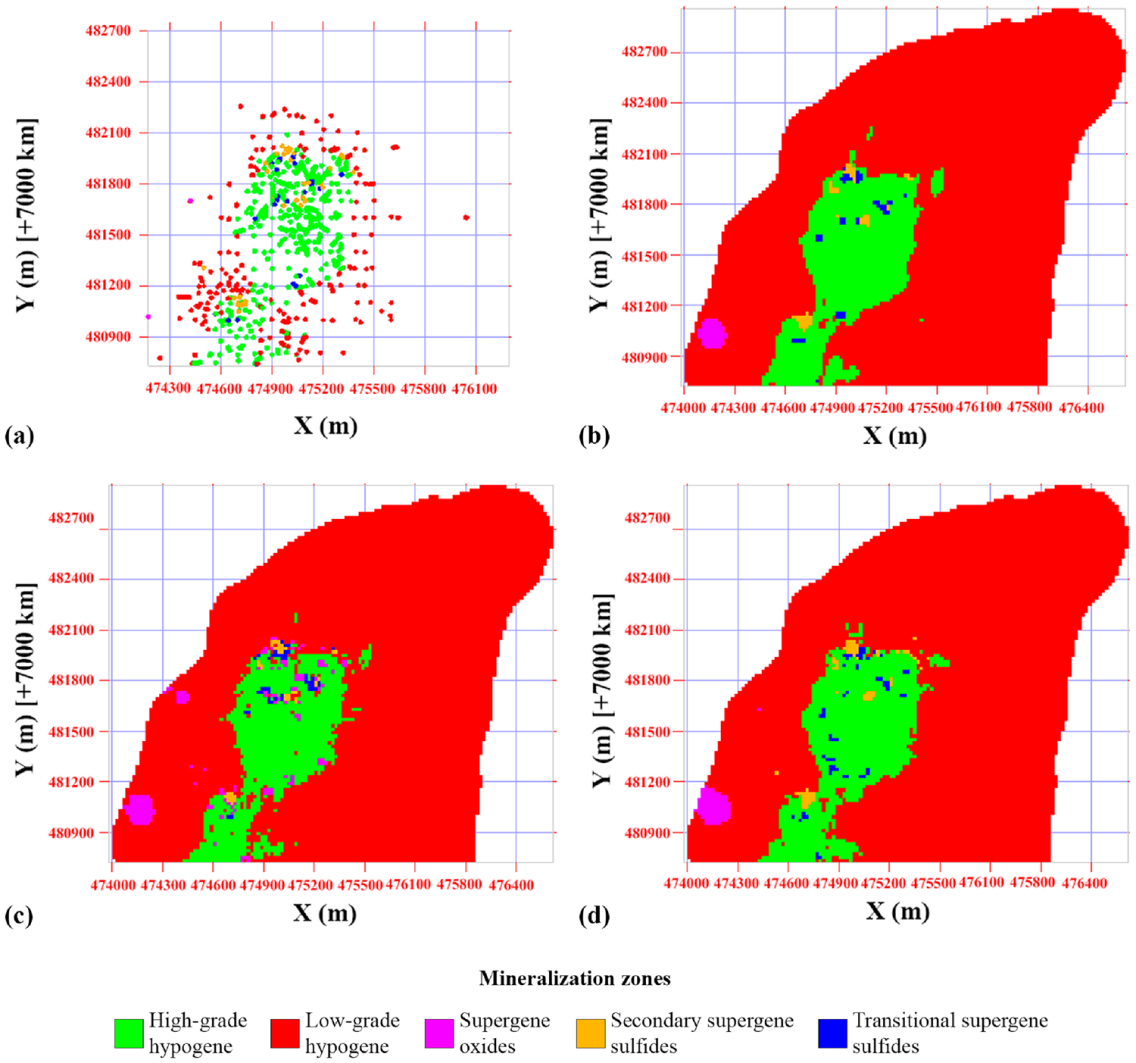
**(a)** Locations of drill hole data, **(b)** Interpreted model of mineralization zones, and most probable mineralization zones calculated from 100 realizations generated by **(c)** Implicit Boundary Simulation and **(d)** Hierarchical Plurigaussian Simulation. Plan views at elevation 1522*m*.

Discrepancies are further evident in the maps of the most probable mineralization zones derived from each approach (Fig. 15c and d). Notably, the Implicit Boundary Simulation exhibits greater deviations from the interpreted mineralization model compared to Hierarchical Plurigaussian Simulation. For instance, in the interpreted model, the central part of the study area is predominantly classified as high-grade hypogene, with no occurrence of supergene oxides. However, the most probable mineralization map produced by Implicit Boundary Simulation classifies several blocks in this region as supergene oxides, which is inconsistent with both the interpreted model (Fig. 15b) and drill hole data (Fig. 15a). This discrepancy between Implicit Boundary Simulation and the interpreted model is particularly pronounced in the central part of the study area, where drill hole data are abundant, and all mineralization zones coexist. In this context, the accuracy of signed distance calculations may be reduced due to the high variability of mineralization zones over short spatial scales, as reflected in the drill hole data. In fact, the analysis of consecutive data along the same drill hole revealed that, at this specific elevation, mineralization zones were occasionally interwoven over a scale of a few meters.

To complement the visual analysis, Tables 2 and 3 summarize the matches and mismatches between the interpreted mineralization model and the most probable mineralization zones derived from Implicit Boundary Simulation and Hierarchical Plurigaussian Simulation.

The results indicate that the consistency between Implicit Boundary Simulation and the interpreted model is 90% for high-grade hypogene, 97% for low-grade hypogene, 49% for secondary supergene sulfides, and 88% and 43% for supergene oxides and transitional supergene sulfides, respectively. In contrast, Hierarchical Plurigaussian Simulation achieves consistencies of 89%, 98%, 86%, 86%, and 74% for the same mineralization zones. These findings suggest that the latter approach achieves greater consistency with the interpreted geological model, particularly for supergene oxides and transitional supergene sulfides, indicating a stronger integration of the imposed geological constraints within the simulation process. Both approaches are successful in modeling dominant mineralization zones, such as high-grade and low-grade hypogene. However, Implicit Boundary Simulation exhibits limitations in realistically modeling disseminated domains, which are discontinuous and occupy a smaller proportion of the study area. As previously discussed, this limitation may stem from the challenges in accurately computing signed distances in regions with high variability in mineralization zones.

**Table 2:** Matches and mismatches between the interpreted block model and the model with the most likely mineralization zones according to Implicit Boundary Simulation.

Most probable mineralization zone	Interpreted mineralization model				
	High-grade hypogene	Low-grade hypogene	Supergene oxides	Secondary supergene sulfides	Transitional supergene sulfides
High-grade hypogene	4141	596	390	44	185
Low-grade hypogene	227	39,873	406	43	83
Supergene oxides	0	166	1170	0	0
Secondary supergene sulfides	87	320	308	1324	84
Transitional supergene sulfides	127	77	112	87	266

**Table 3:** Matches and mismatches between the interpreted block model and the model with the most likely mineralization zones according to Hierarchical Plurigaussian Simulation.

Most probable mineralization zone	Interpreted mineralization model				
	High-grade hypogene	Low-grade hypogene	Supergene oxides	Secondary supergene sulfides	Transitional supergene sulfides
High-grade hypogene	4925	265	0	104	63
Low-grade hypogene	234	40,145	184	66	3
Supergene oxides	0	163	1172	1	0
Secondary supergene sulfides	133	185	0	1757	48
Transitional supergene sulfides	209	14	0	107	339

## Discussion

Despite the fundamental differences in the underlying principles of these two approaches, they share certain commonalities that influence the results. Both methods rely on an interpreted geological model to address data scarcity and account for non-stationary domains (spatial zonations). Consequently, the quality and reliability of the interpreted geological model play a crucial role in determining the accuracy of the outcomes. To ensure robustness, the interpreted model should be constructed with a high degree of confidence, integrating diverse data sources such as geological maps, cross-sectional exploration data, conceptual lithological and mineralogical profiles, geological logs from drill holes, and expert geological knowledge. Another key factor influencing the accuracy of both approaches is the discrepancy between the interpreted geological model and drill hole data. Inconsistencies arise in regions where drill hole data indicate the presence of a specific domain, while the interpreted model assigns a different domain to the same location. These inconsistencies significantly impact the results. To mitigate this issue, preliminary data cleaning can be conducted to reduce discrepancies and improve accuracy. Additionally, both approaches require careful reproduction of geological relationships (e.g., stratigraphic sequences and faulting) in the simulation process. This necessitates a precise definition of the hierarchical binary tree in Implicit Boundary Simulation and the truncation tree in Hierarchical Plurigaussian Simulation, both of which are inherently subjective and heavily reliant on expert judgment.

From an implementation perspective, Implicit Boundary Simulation appears to be more straightforward, as calculating signed distances between samples and domain boundaries is generally simple, particularly when geological domains are well-defined. However, this process becomes increasingly complex in areas with sparse data or high geological variability, where defining boundaries is challenging and associated with significant uncertainty. A critical challenge arises when estimating distances for drill holes that do not intersect geological boundaries, meaning the entire drill hole remains within a single mineralization zone. In such cases, distance estimations introduce additional uncertainty into the results. Furthermore, calculating the shortest down-the-hole distance to a boundary is only feasible when data are available in a drill hole format. If the data are provided as scattered points in space rather than structured drill hole information, down-the-hole distance calculations become impractical, and an omnidirectional distance approach must be used instead.

From a computational standpoint, Hierarchical Plurigaussian Simulation is more demanding compared to Implicit Boundary Simulation, primarily due to the Gibbs sampling step required to transform the data coding the domains into Gaussian data. This step is computationally intensive and sensitive to convergence criteria, contributing to increased computational costs. Table 4 compares and summarizes the two approaches, based on the results obtained from the constructed case study, across various aspects.

**Table 4:** Comparison of implicit boundary simulation and hierarchical Plurigaussian simulation based on the results obtained from this study.

	Implicit boundary simulation	Hierarchical Plurigaussian simulation
Incorporation of interpreted model	Incorporated via a co-simulated auxiliary signed distance ($V_2$)	Used to estimate local domain proportions that guide truncation thresholds and indicator-to-Gaussian transformations
Control of soft data influence	Controlled by a confidence coefficient (*ρ*) applied to the sill of the cross-variogram	Controlled by the size of the moving window used to calculate local domain proportions
Computational demand	Moderate; each level simulated separately	Higher; especially due to the Gibbs sampler algorithm
Computational time per realization$^{*}$	*≈* 40 seconds	*≈* 80 seconds
Sensitivity to parameter selection	Highly sensitive to *ρ*; incorrect choice can lead to overfitting or under-utilization of soft data	Highly sensitive to moving window size; wrong choice can overly mimic the model or fail to capture local trends
Inference parameters	Requires fewer and simpler inference parameters (primarily the calculation of signed distances)	Requires more complex inference parameters, including the derivation of Gaussian variograms from experimental indicator variograms
Flexibility for complex contacts	Less flexible; better for nested or stratified domains	More flexible; suited for domains with multiple mutual contacts

$^{*}$Hardware: Intel(R) Xeon(R) Silver 4208 CPU @ 2.10GHz, 32.0 GB RAM (2400 MT/s), NVIDIA T400 GPU (2 GB). Number of conditioning drill hole data: 32,924; number of secondary data: 50,116; number of locations targeted for simulation: 50,116.

In the authors’ opinion, IBS may be preferable in geological scenarios where drill hole data are scarce but a geologically coherent interpreted model is available. Under such conditions, the variogram of the latent random field $Y_1$ inferred from the drill hole data alone is often unreliable. In contrast, IBS partially overcomes this limitation by incorporating an auxiliary random field $Y_2$, whose spatial continuity can be inferred reliably from the interpreted block model. When $Y_2$ is strongly correlated with $Y_1$, this additional information helps inferring the bivariate variogram model and improves domain simulation in poorly sampled areas.

On the contrary, HPGS is more appropriate when there is significant inconsistency between the interpreted geological model and the drill hole observations, in particular, when the interpreted model exhibits much smoother domain boundaries than the drill hole data. In IBS, such inconsistencies can affect the modeling of the direct and cross variograms of $Y_1$ and $Y_2$, especially at small distances (classical coregionalization models cannot handle variables with a different degree of smoothness), and reduce the robustness of the simulation framework. In HPGS however, the variograms of the latent Gaussian random fields are inferred directly from the drill hole data and do not involve the interpreted geological model, making the modeling of the spatial continuity less sensitive to the latter model.

Therefore, from a practical standpoint, IBS is recommended when the objective is to explicitly integrate reliable soft geological information into the simulation process in early exploration stages when hard data are scarce. HPGS is recommended when the priority is to reproduce hard-data-driven spatial continuity with less dependence on an interpreted geological framework.

## Conclusions

In this study, Implicit Boundary Simulation and Hierarchical Plurigaussian Simulation were applied to model mineralization zones in a porphyry copper deposit under conditions of sparse sampling. Both approaches incorporate and rely on an interpreted mineralization model, constructed based on geological knowledge of the deposit, as a secondary source of information to better capture and reproduce the spatial distribution of mineralization zones. In each approach, the interpreted model is incorporated in a different way. In Hierarchical Plurigaussian Simulation, the interpreted model is used to calculate the local domain proportions and, consequently, the local truncation thresholds, instead of considering global proportions. In Implicit Boundary Simulation, the interpreted model is applied as a covariate that is cross-correlated with the primary data. The influence of the interpreted model on the final results can be controlled by adjusting the size of the moving window and by reducing the cross-correlation between drill hole data and the interpreted model in Hierarchical Plurigaussian Simulation and Implicit Boundary Simulation, respectively. Additionally, subjectivity in defining hierarchical structures, such as the truncation tree in Hierarchical Plurigaussian Simulation and the binary tree in Implicit Boundary Simulation, influences the outcomes and requires careful expert judgment (reliance on a geological conceptualization of the mineral deposit).

The results indicate that, while both methods yield generally similar outcomes, Hierarchical Plurigaussian Simulation produces representations of mineralization zones that are more consistent with the interpreted model, particularly for the supergene oxides and transitional supergene sulfides. Overall, in Implicit Boundary Simulation, the modeling is less consistent in areas with high short-scale variability in mineralization zones, as observed in drill hole data. This limitation is primarily due to challenges in accurately computing signed distance fields in such geologically complex regions.

## Data Availability

The datasets and codes generated and/or analyzed during the current study are not publicly available due to confidentiality restrictions but are available from the corresponding author upon reasonable request.
